# Do 'alternative' help-seeking strategies affect primary care service use? A survey of help-seeking for mental distress

**DOI:** 10.1186/1471-2458-8-207

**Published:** 2008-06-11

**Authors:** Katja Rüdell, Kamaldeep Bhui, Stefan Priebe

**Affiliations:** 1 Affiliation at time of research, Centre for Psychiatry, Barts & the London School of Medicine, Queen Mary University of London, UK; 2 Outcomes Research, Pfizer Ltd (IPC 160), Sandwich, Kent, CT13 9 NJ, UK

## Abstract

**Background:**

Epidemiological studies suggest that only some distressed individuals seek help from primary care and that pathways to mental health care appear to be ethnically patterned. However few research studies examine how people with common mental disorder manage their mental distress, which help-seeking strategies they employ and whether these are patterned by ethnicity? This study investigates alternative help-seeking strategies in a multi-ethnic community and examines the relationship with primary care use.

**Methods:**

Participants were recruited from four GP practice registers and 14 community groups in East London. Of 268 participants, 117 had a common mental disorder according to a valid and structured interview schedule (CIS-R). Participants were of Bangladeshi, black Caribbean and White British ethnic background. For those with a common mental disorder, we examined self-reported help-seeking behaviour, perceived helpfulness of care givers, and associations with primary care service use.

**Results:**

We found that alternative help-seeking such as talking to family about distress (OR 15.83, CI 3.9–64.5, P < .001), utilising traditional healers (OR 8.79, CI 1.98–38.93, p = .004), and severity of distress (1.11, CI 1.03–1.20, p = .006) was positively associated with primary care service use for people with a common mental disorder. Ethnic background influenced the choice of help-seeking strategies, but was less important in perceptions of their helpfulness.

**Conclusion:**

Primary care service use was strongly correlated with lay and community help-seeking. Alternative help-seeking was commonly employed in all ethnic groups. A large number of people believed mental distress could not be resolved or they did not know how to resolve it. The implications for health promotion and integrated care pathways are discussed.

## Background

The incidence and prevalence of mental health problems is a growing concern for public health worldwide [1]. Epidemiological studies across different nations show that individuals rarely access and use health services for mental distress [2-6]. Gender, age, minority ethnic background, severity of symptoms and physical co-morbidity, as well as mental health awareness, concerns about the effects of a diagnosis and doubts about the effectiveness of medication are all potential barriers to service use [5,7-12]. The 'community' can be an important resource in caring for the mentally distressed [13,14]; using such resources may deter individuals from using conventional mental health and primary care services [15]. On the other hand, lay networks may prevent suicide and can positively influence outcome and mental health service use [16-18]. In North America, studies have explored influences on intentions to seek care; patients' views of their doctor's interpersonal skills as well as their views of their own illnesses were both important in the decision to seek formal help [19,20]. Although there is some recognition of the importance of community and alternative lay help-seeking patterns [14,21,22], there is little information about the type and range of coping strategies and sources of help sought by people with common mental disorders.

In multi-ethnic communities, the use of an alternative economy in form of 'traditional' healing has been described [23,24]. The WHO described 'traditional medicine [as] the knowledge, skills and practice of holistic health care, recognized and accepted for its role in the maintenance of health and the treatment of diseases. It is based on indigenous theories, beliefs and experiences that are handed down from generation to generation' [25]. Ahmed et al suggest that 'A traditional healer is defined as an educated or lay person who claims to have ability or a healing power to cure ailments, or a particular skill to treat specific types of complaints or afflictions and who might have gained a reputation in his own community or elsewhere. They may base their powers or practice on religion, the supernatural, experience, apprenticeship or family heritage.' [26]. Although a WHO survey and anthropological studies suggest that use of traditional healers is widespread [27], no surveys appears to have explored whether this practice deters people from using medical services in primary care.

This paper considers a range of strategies (behavioural, social, spiritual etc) to manage common mental disorder and the associated mental distress. These help seeking strategies include seeking care from lay people, traditional healers, community agencies and primary care. The research aims are: to examine the pattern of help-seeking strategies in response to experiences of mental distress, to explore people's views on the helpfulness of medical services and finally to determine whether use of alternative services correlates with less use of medical services. In particular, we wanted to answer the following questions:

Firstly, 1a) when people experience mental distress what help-seeking strategies do they try and which are perceived helpful?; 1b) is the use and preferences for 'help-seeking' strategies ethnically patterned, if so – are there specific types of help-seeking (medical, alternative, spiritual etc) that certain groups prefer? Finally, 1c) are there differences between ethnic groups in how many people are approached for help?

Our second research aim concerned the experience of primary care services as reported by mentally distressed people. We wanted to know 2) what people find helpful/unhelpful when they talk to their GPs? Our final question was: 3) do alternative help-seeking strategies affect individuals' use of primary care services negatively?

## Methods

### Setting

The survey was called 'perceptions about stress, distress and illness' and took place in 4 general practices and 14 community organisation in East and South London where there is a higher concentration of these ethnic groups. Most of the data (80%) was collected in Hackney and Tower Hamlets, 2 of the most deprived boroughs in the UK. The local NHS research ethics committee approved the study (REF # Do/SG/02/145).

### Participants

Details of all participant characteristics have been published elsewhere [28,29]. We used a mixed method sampling frame in order to ensure a wide range of people from different demographic backgrounds were represented. As part of our sampling frame we used four general practice registers and randomly sampled 871 Black Caribbean, Bangladeshi or White British individuals using ethnic codes recorded in the notes. We sent all individuals invitations to participate in a survey about perceptions of illness, stress and distress: 29%, 44% & 46% of the respective ethnic group agreed to participate. We attempted to contact everyone who had not opted out via telephone, which revealed that 393 records (46%) were inaccurate for reasons such as incorrect details including missing ethnic codes and residential re-location without a follow up phone number. Response rates among those with correct contact information were 43%, 56% and 64% respectively. We supplemented our reactive sampling from GPs with pro-active sampling from community groups. Reactive sampling has been described as reactive to individuals' responses to invitations; proactive sampling actively seeks recruitment into studies e.g. by 'door-knocking' randomly approaching houses in a given community [30]. We proactively recruited attendees of non-health community sites including colleges, community groups, and social venues from a wide range of ages and demographic strata. Just under half the final sample was recruited from GP services (43.9%; community agencies 56.1%).

There were no significant differences between the reactive and proactive samples on a number of characteristics: age, number of children, length of stay in the UK in years, gender, place of birth, occupation and number of first generation individuals. Significant differences were found between the 2 samples in terms of further education and accommodation – the proactive sample from community sites had higher level of further education (Chi Square = 5.17, p = .023); and lower level of owned accommodation than the reactive sample from the GP surgeries (Chi Square = 36.5, p < .0001). Ethnic background was confirmed at interview. No enticements were given to participate.

We recruited 365 participants for the survey overall (125 White British, 116 Black Caribbean, and 122 Bangladeshi people) and of these 268 (73.4%) reported that something distressing had happened to them in the past month [28,29]. Of these 117 (43.7%) met criteria for common mental disorder (score of 12 or above on a standard psychiatric diagnostic interview (Clinical Interview Schedule – Revised [31]) These form our sample of people with common mental disorder; the socio-demographic characteristics are set out in article Table 1.

**Table 1:** Characteristics (social, ethnic and psychological factors) of cases

Demographic Characteristics		White British (n = 39)	Caribbean (n = 21)	Bangladeshi (n = 57)	Statistical test Χ^2 ^& KW
Age range (y)	Mean	38.1	41.7	43.1	KW 1.86 (2), p = .39
Gender	Female	71.8%	85.7%	50.9%	9.60 (2), p < .01
Place of Birth	UK	92.3%	47.6%	5.3%	149.16 (6), p < .001
Length of stay in UK (y)	Mean		27.7	19.6	KW 2.73 (2), p = .25
Recruitment Venue	GP	61.5%	38.1%	47.4%	3.41 (2) p = .18
Primary education in the UK	Yes	89.5%	66.7%	19.0%	41.40 (2), p < .001
Number of Children	Mean	0.8	1.9	2.9	24.15, p < .001
Employment status	Paid Employment	46.2%	19%	12.4%	20.61 (4), p < .001
	Homemaker	12.8%	0	23.6%	
Receives Benefits	Yes	47.2%	52.4%	77.3%	10.28 (2), p < .01
Accommodation	Owned	39.4%	14.3%	26.8%	8.97 (4), p = .06
	Rented	20.5%	23.8%	7.1%	
Chronic Physical Illness	Yes	68.4%	76.2%	75.4%	.76, p = .68
Clinical Interview Schedule – Revised	Mean	20.6	21.9	21.0	KW.34 (2), p = .84

### Procedure

Following informed consent, and confirmation of the ethnic group, we administered the survey in the individuals' chosen language (English for White British and Black Caribbean people and Sylheti – a Bangladeshi dialect spoken in the area of Sylhet, or standard Bengali or English for Bangladeshi participants). All interviews were taped and some translated from Bengali/Sylheti where necessary.

We used the Barts Explanatory Model Inventory (BEMI). This was developed to capture individuals own representation or explanatory model of distress [29] on five domains: identity, cause, timeline, consequences and control/cure [32,33]. Help-seeking behaviours are assessed in the BEMI as part of the control/cure domain. The BEMI consists of two perception assessments, the first a semi-structured qualitative interview protocol (BEMI-I) and the second a perception and help-seeking strategy checklist (BEMI-C). The BEMI-I immediately precedes the BEMI-C and the two are complementary. In the help-seeking section of the BEMI-I, open ended questions are included to explore how distress can be best resolved; who has been talked to about their distress; whether this was seen as helpful and why this was so. The BEMI-I assesses participants' help-seeking strategies freely without any constraints thereby reducing the likelihood of response bias. After all open-ended data were transcribed; two researchers independently coded the data generating categories that most parsimoniously enabled all the date to be coded. Disagreements on the narratives about help-seeking were discussed by these two researchers until agreement was reached. At each stage, strategies were identified as a new category if they did not easily fit within the emergent range of categories. The help seeking section of the BEMI-C asks individuals to rate 18 help-seeking strategies regarding their current distressing episode. Participants were asked to indicate whether they considered or used different strategies. If they did use them they were asked whether they found these helpful.

### Data Coding

The interviews were conducted by five independent researchers (two sociology students, two psychologists and a medical doctor) trained in the administration of the CIS-R and BEMI. Interrater reliability was established prior to data collection as adequate (Kappa >0.8). Interviews with individuals from ethnic minorities were conducted by ethnically and language matched interviewers.

## Results

### Aim 1a) What help-seeking strategies do people use and find helpful? (BEMI-I and BEMI-C, Table 2)

**Table 2:** Use and Helpfulness of Help-seeking Strategies

Themes	BEMI-I What is the best way to resolve or deal with your distress? n (%) (n = 117)	BEMI-C Have you tried the strategy to resolve your distress? n (%) (n = 113)^a^	BEMI-C Have you found the strategy helpful to resolve your distress? n (%) (n = 113)^a^
Self Directed help-seeking strategies			
Keeping Busy	1 (.9%)	72 (63.7%)	45 (39.8%)
Exercise	1 (.9%)	67 (59.3%)	41 (36.3%)
Dieting/Fasting	2 (1.7%)	47 (41.6%)	20 (17.7%)
Thinking	0	42 (37.2%)	21 (18.6%)
Using alcohol, drugs	0	38 (33.6%)	13 (11.5%)
Spending time on a hobby	1 (.9%)	29 (25.7%)	21 (18.6%)
Dancing	0	21 (18.6%)	17 (15.0%)
Chanting	0	18 (15.9%)	10 (8.8%)
Overall endorsement of self directed strategies % of individuals who used 1 or more		102 (90.3%)	78 (69.0%)
			
Social help-seeking strategies			
Talk to your friends	4 (3.4%)	71 (62.8%)	49 (43.4%)
Talk to your family	3 (2.6%)	71 (62.8%)	45 (39.8%)
Socialising	2 (1.7%)	65 (57.5%)	44 (38.9%)
Overall endorsement of social strategies % of individuals who used 1 or more		96 (85%)	75 (66.4%)
			
Spiritual help-seeking strategies			
Praying	1 (.9%)	47 (41.6%)	30 (26.5%)
			
Medical help-seeking strategies			
Talk to your GP	6 (5.1%)	53 (46.9%)	29 (25.7%)
Taking medication	9 (7.7%)	50 (44.2%)	31 (27.4%)
Overall endorsement of medical strategies % of individuals who used 1 or more		68 (60.2%)	47 (41.6%)
			
Alternative help-seeking strategies			
Using herbal remedies	1 (.9%)	39 (34.5%)	21 (18.6%)
Relaxation/massage	4 (3.4%)	33 (29.2%)	25 (22.1%)
Seeing a traditional healer	1 (.9%)	22 (19.5%)	11 (9.7%)
Yoga/meditation	2 (1.7%)	11 (9.7%)	5 (4.4%)
Overall endorsement of alternative strategies % of individuals who used 1 or more		64 (56.6%)	38 (33.6%)

NB

a Total participant numbers differed due to missing data on BEMI checklists.

Overall percentages are higher than 100% as multiple answers were possible

Table 2 displays help-seeking strategies on both BEMI-I and BEMI-C for the overall sample.

In the BEMI-I, individuals were asked how their distressing experiences could be best resolved. Thirty nine themes emerged in these narratives. The most common response was no idea/don't know (28 participants; 23.9%). This answer was significantly more common among ethnic minorities (22.3% BA; 25.6%BC; 9% WB; Chi Square -10.83, p = .004). The second most common strategy was to resolve circumstances such as housing, management of illness/or pregnancy mentioned (21 participants; 17.9%). The third most common answer was that no resolution was possible (17; 14.5%). Other ways of resolving distress were better communication (13; 11.1%), personal resilience/coping (12; 10.3%), and taking more ownership of their lives (10; 8.5%) and getting a job or changing a job (10; 8.5%)

In BEMI-C, the most commonly tried strategy for distress was 'keeping busy', closely followed by 'talking to family', 'talking to friends' and 'exercise'. We also asked all individuals which strategy they found helpful; 'talking to friends' was the most often used strategy closely followed by 'talking to family' and 'keeping busy', 'socialising' and 'exercise'.

### 1b) Is the use of and preference for 'help-seeking' strategies ethnically patterned, if so – are there specific types of help-seeking (medical, alternative, spiritual etc) that certain groups prefer? (BEMI-C; Table 3, 4 &5)

**Table 3:** Ethnic differences in the use of help seeking strategies (BEMI-C)

Questionnaire Survey		White British	Black Caribbean	Bangladeshi	Used Strategy Χ^2^(df)	Used Strategy p-value
Self-directed Help-seeking Behaviour	Exercising	24 (66.7%)	12(60.0%)	31 (54.4%)	1.38 (2)	p = .50
	Changing your diet	18 (50.0%)	8 (40.0%)	21 (36.8%)	1.60 (2)	p = .45
	Using drugs or alcohol	21(58.3%)	8 (40.0%)	9 (15.8%)	18.33 (2)	P < .001
	Keeping busy	24 (66.7%)	13 (65.0%)	35 (61.4%)	.28 (2)	p = .86
	Chanting	3 (8.3%)	2 (10.0%)	13 (22.8%)	4.09 (2)	p = .13
	Dancing	11 (30.6%)	8 (40.0%)	2 (3.5%)	18.03 (2)	P < .001
	Thinking	19 (52.8%)	8 (40.0%)	15 (26.3%)	6.70 (2)	P = .035
	Spending time on a Hobby	16 (44.4%)	7 (35.0%)	6 (10.5%)	14.42 (2)	P < .001
						
Social help-seeking	Talking to your family	23 (63.9%)	9 (45.0%)	39 (68.4%)	3.50 (2)	p = .17
	Talking to your friends	27 (75.0%)	12 (60.0%)	32 (56.1%)	3.44 (2)	p = .18
	Socialising	23 (63.9%)	9 (45.0%)	33 (57.9%)	1.88 (2)	p = .39
						
Spiritual Help seeking	Praying	6 (16.7%)	13 (65.0%)	28 (49.1%)	15.05 (2)	P < .001
						
Medical Help seeking	Talking to your GP	13 (36.1%)	10 (50.0%)	30 (52.6%)	2.51 (2)	p = .29
	Taking Medication	11 (30.6%)	5 (25.0%)	34 (59.6%)	11.22 (2)	P = .004
						
Complementary Service Use	Using herbal remedies	16 (44.4%)	12 (60.0%)	11 (19.3%)	13.16 (2)	P < .001
	Relaxation and massage	14 (38.9%)	9 (45.0%)	10 (17.5%)	7.80 (2)	p = .02
	Yoga	7 (19.4%)	3 (15.0%)	1 (1.8%)	8.62 (2)	P = .013
	Seeing a traditional healer	3 (8.3%)	3 (15.0%)	16 (28.1%)	5.79 (2)	P = .055

**Table 4:** Ethnic differences in ratings of help seeking strategies as helpful (BEMI-C)

Questionnaire Survey		White British	Black Caribbean	Bangladeshi	Helpful Strategy Χ^2^(df)	Helpful Strategy p-value
Self-directed Help-seeking Behaviour	Exercising	16 (66.7%)	9 (75.0%)	16 (51.6%)	2.46 (2)	p = .29
	Changing your diet	6 (33.3%)	8 (100%)	6 (28.6%)	13.10 (2)	p < .001
	Using drugs or alcohol	5 (23.8%)	3 (37.5%)	5 (55.6%)	2.87 (2)	p = .23
	Keeping busy	13 (54.2%)	9 (69.2%)	23 (65.7%)	1.11 (2)	p = .57
	Chanting	1 (33.3%)	2 (100%)	7 (53.8%)	2.22 (2)	p = .33
	Dancing	8 (72.7%)	8 (100%)	1 (50.0%)	3.61 (2)	p = .17
	Thinking	9 (47.4%)	8 (100%)	4 (26.7%)	11.32 (2)	p = .003
	Spending time on a Hobby	12 (75.0%)	7 (100%)	2 (33.3%)	7.31 (2)	p = .026
						
Social help-seeking	Talking to your family	14 (60.9%)	8 (88.9%)	24 (61.5%)	3.58 (2)	p = .16
	Talking to your friends	18 (66.7%)	11 (91.7%)	20 (62.5%)	5.54 (2)	p = .06
	Socialising	14 (60.9%)	9 (100%)	21 (63.6%)	5.03 (2)	p = .08
						
Spiritual Help seeking	Praying	2 (33.3%)	10 (76.9%)	18 (64.3%)	3.39 (2)	p = .18
						
Medical Help seeking	Talking to your GP	6 (46.2%)	8 (80.0%)	15 (50.0%)	3.23 (2)	p = .19
	Taking Medication	4 (36.4%)	4 (80.0%)	23 (67.6%)	4.21 (2)	p = .12
						
Complementary Service Use	Using herbal remedies	10 (62.5%)	7 (58.3%)	4 (36.4%)	1.93 (2)	p = .38
	Relaxation and massage	12 (85.7%)	8 (86.9%)	5 (50.0%)	5.21 (2)	p = .07
	Yoga	4 (57.1%)	1 (33.3%)	0	1.40 (2)	p = .50
	Seeing a traditional healer	3 (100%)	3 (100%)	5 (31.3%)	8.25 (2)	p = .016

**Table 5:** Ethnic differences in the use of strategies (BEMI-C) and people approached for help (BEMI-I)

Strategies BEMI-C*	White British (n = 39)	Black Caribbean (n = 21)	Bangladeshi (n = 57)	Χ^2a ^& KW
Range	2–16	0–18	0–14	KW 2.86; p = .24
Mean	7.75	7.55	6.42	
				
Self Directed (Max 8)	3.78	3.03	2.31	KW 11.24; p = .004
(% of at least 1 strategy)	100%	85.0%	86.0%	Χ^2 ^= 33.03; p <.007
				
Social (Max 3)	2.03	1.50	1.83	KW 2.67; p =.26
(% of at least 1 strategy)	94.4%	75.0%	82.5%	Χ^2 ^= 5.47; p =.49
				
Spiritual (Max 1)	.17	.65	.49	KW 14.91; p =.001
(% of at least 1 strategy)	16.7%	65.0%	49.1%	Χ^2 ^= 15.05; p =.001
				
Medical (Max 2)	.67	.75	1.12	KW 6.96; p =.031
(% of at least 1 strategy)	52.8%	55.0%	66.7%	Χ^2 ^= 12.03; p =.017
				
Complementary (Max 4)	1.11	1.35	.67	KW 8.80; p = .012
(% of at least 1 strategy)	58.3%	85.0%	45.6%	Χ^2 ^= 15.16; p =.056
				
Number of people consulted (BEMI I)				
Range	0–9	0–5	0–5	
Mean	3.33	1.95	1.95	KW 10.36, p < .01
% who contacted at least 1	84.6%	66.7%	77.2%	Χ^2 ^= 22.28; p =.13

* Participant numbers varied for BEMI-C WB = 36 BC = 20,

^a ^Χ^2 ^tests the distribution between the whole range of methods, but we have presented percentages of different ethnic groups using at least 1 strategy per domain

The use of help-seeking strategies was ethnically patterned as significant differences were found in these strategies: 'Substance use', 'dancing', 'thinking' 'spending time on a hobby', 'praying', 'taking medication',' using herbal remedies', 'relaxation', 'yoga' and marginally 'seeing a traditional healers'. Fewer ethnic differences were found in strategies that were considered as helpful: 'changing your diet', 'thinking', 'spending time on a hobby' and 'seeing a traditional healer'.

The survey found that among all ethnic groups, the Bangladeshi group had the highest proportion of individuals who sought help from their GP; the black Caribbean group had the highest proportion who found talking to their GP helpful. Bangladeshi subjects were most likely to receive medication and Caribbean and Bangladeshi subjects were most likely to find that helpful. Whereas a larger proportion of Bangladeshi participants saw traditional healers, fewer of them found this helpful. Black Caribbean subjects were least likely to talk to family or friends about their distressing experience, but a higher proportion of those who did rated this as helpful compared to the other ethnic groups.

We aggregated all strategies according to categories (e.g. self-directed, social, spiritual, medical, alternative) and tested for ethnic preferences among these. The validity and the reliability of these categories were established in the development of the BEMI in early work [29]. We tested whether ethnic background affected the mean number of strategies used and whether it affected the uptake of at least 1 strategy of each type.

All individuals preferred self-directed strategies independent of their ethnic background. However particular themes of help-seeking strategies were more popular in some groups than others (See Table 5). A higher proportion of White British participants used self-directed and social help-seeking strategies, whereas they were less likely to report spiritual help-seeking than the other groups. Complementary help-seeking was particularly common amongst White British and Black Caribbean participants, but not as important for Bangladeshi participants who in turn favoured medical strategies more than the other ethnic groups.

Experience of other health care sectors might also affect people's help-seeking choices. Therefore, we were interested to unravel whether exposure to health care and age at migration affected these results. We compared migrants with non-migrants. Only 113 subjects gave information about their migration status. 40 had not migrated at all, 27 migrated as children (below the age of 18) and 48 as adults. Non-migrants included 28 White British, 10 Black Caribbean and 2 Bangladeshi subjects. Child migrants included 4 Black Caribbean 15 Bangladeshi and 8 White British subjects. Adult migrants comprised 36 Bangladeshi, 7 Black Caribbean, and 3 White British subjects.

Self help and alternative treatment were preferred by non-migrants and individuals who had migrated as children compared with adult migrants (KW = 7.19, p = .027; KW = 6.44, p < .04). Social treatments were most preferred by migrants who had migrated as children, those who had not migrated, and least by those who migrated as adults (KW = 6.28, p = .043). Spiritual treatments were most commonly sought by those who had migrated as children closely followed by those who had migrated as adults, but not as much by individuals who had not migrated (KW = 9.62, p = .006).

### 1c) Does ethnic group membership influence the number of people consulted? (BEMI I, Table 4)

Ethnic group membership was also significantly associated with the number of people consulted about the distressing experience. White British subjects talked to significantly more people than the two other groups which did not differ significantly from each other. We also tested whether the distribution of people who talked to no one differed from those who talked to at least one person; there was no significant difference between ethnic groups.

### 2) When people see their GP about distress, what do they find helpful and what unhelpful? (BEMI-I)

We asked individuals why they found talking to someone helpful (or not helpful) within the BEMI-I. We present some of the data captured as 'narrative' for those who did talk to their GP; we wanted to discern whether subjects found consultations helpful.

Talking to GPs was not helpful when the doctor was unresponsive to subjects' worries and concerns:

Male Caribbean, 68 years, Retired: "The doctor just says the same thing;"

Female Caribbean, 74 years Retired: "Not helpful to talk to Dr, because s/he gives the same answer 'it takes time';"

Male Bangladeshi, 52 years: "They are not taking my problem seriously."

GPs were also viewed as unhelpful when they appeared too focussed on the medication:

Female White British, 29 years: "Doctor did not offer solution, but medication;"

Male White British, 37 years: "GP was a bit off-hand, [his/her treatment recommendations] didn't work, they don't like that and give you some pills. Maybe [they were] just busy, but that is dangerous, I really was a bit desperate."

In one case, a respondent said that the GP was not helpful since the situation was hopeless:

Male Bangladeshi, 31 years: "What's inside me cannot be taken out. They can't help me, no one can. Only I can help myself by killing myself."

Those individuals who viewed the GP service as helpful said that the treatment had worked for them:

Male Bangladeshi, 62 years: "GP has given me medicine, I feel a bit better now."

Patients that rated primary care services as helpful were also more aware of the constraints that GPs were working with and had lower expectations of the service:

Female Caribbean, 36 years: "Some doctors don't have time. 10 min is not enough time, A doctor who understands my lifestyle and is acknowledging that is enough"; Female White British, 36 years: "GP referred to the top route [which] was very good."

### 3) Are alternative help-seeking strategies associated with individuals' primary care use?

Gender, age, ethnicity and co-morbid physical illness as well as severity of distress, help seeking from family, friends and traditional healers were entered in a logistic regression that analysed associations with primary care service use.

The results are presented in Table 6. In adjusted analyses, primary care services were more likely to be used by people who had greater severity of distress (OR (per score point on CIS-R): 1.11, 1.03–1.20, p = .006), sought help from family (OR 15.83, 3.89–64.48, p = .0001) or traditional healers (OR 8.79, 1.98–38.94, p = .004).

**Table 6:** Logistic Regression Analyses predicting primary care service use

	Cases	N = 114	
			
Logistic Regression analyses	Odds ratio	CI 95%	p value
Age	1.03	.99–1.07	.138
Belonging to an ethnic minority	.58	.18–1.86	.364
Gender	.51	.16–1.65	.260
Comorbidity (Physical illness	2.16	.65–7.27	.210
Severity of distress (CIS-R Score)	1.11	1.03–1.20	.006
Sought help from family (BEMI-C)	15.83	3.89–64.47	.0001
Sought help from friends (BEMI-C)	3.00	.88–10.17	.078
Sought help from Traditional healer (BEMI-C)	8.79	1.98–38.93	.004

## Discussion

Our paper presents findings from a mixed method survey about of the use of help-seeking strategies for dealing with a mentally distressing experience. The findings showed that talking to family and alternative traditional healers were significantly associated with talking to GPs about mental distress. Future research should explore prospective and sequential contacts in care pathways, in order to understand them and develop appropriate interventions targets.

A significant number of people reported not knowing how to resolve their distress; despite this most individuals had tried many strategies and found many alternative strategies helpful. For example, 'Talking to friends', 'family', 'exercising' and 'keeping busy' were viewed as helpful by the majority of distressed individuals.

We also found ethnic differences in the use and preferences for specific help-seeking strategies, and pluralistic help seeking was the norm rather than the exception. Although the use of many strategies was ethnically patterned, there were fewer ethnic differences in what was perceived as helpful. Future research could usefully explore concomitant use of different strategies and their impact on objective measures of outcome. A combination of help-seeking strategies (for example, exercise + medication + herbal remedies) may lead to a different outcome compared with a single strategy or none at all. The health economics of such care pathways also need investigation using objective measures of outcome and measures of recovery.

When administering the BEMI-I, we asked individuals first how could their mental distress or difficulty be best resolved, who they had talked to about their distress and was that helpful?

In the interviews, a quarter said 'no idea' about how to resolve their distress; a sixth preferred social solutions such as changing their environment and social circumstances. A sixth also said that their distress could not be resolved at all. A greater proportion of ethnic minorities were likely not to know who to resolve their distress, reflecting a lack of familiarity with local resources but also perhaps with the notion that mental distress is a condition that health professionals could treat. Many participants reported that the best way to resolve their distress was assistance with concrete problems/circumstances rather than medical treatment. Distressed individuals might therefore appreciate services that support them with practical issues.

Our interview data also identified some strategies that have not been reported previously. These include developing resilience and managing or coping; taking ownership and responsibility, and changing life circumstances or work. These can be assessed in future surveys but also in qualitative work to unravel what people feel constitutes resilience.

The BEMI-C findings showed a high level of endorsement of self-directed and social help-seeking strategies such as talking to family and friends, as well as keeping busy and exercising. These findings are line with other studies. For example Cabassa & Zaya [18] studied help-seeking in Latino immigrants using vignettes; they asked individuals to rank the care providers during the help-seeking; 58% of their sample chose informal care as a first port of call. There were different patterns of help seeking across ethnic groups, with as many as 28.1% of Bangladeshi individuals reported utilising traditional healers (see Table 3). Surprisingly, medical service use and alternative service use were equally common in our survey (see Table 2) suggesting that people may use multiple sources of help, despite reports of a preference for one or other service.

Ethnic differences in the use and preferences for help-seeking strategies point towards specific cultural and individual coping practices which might affect use of services in areas with high level of Bangladeshi or Caribbean participants. This survey also found evidence of changes over time as age at migration affected the use and preference for specific help-seeking strategies. This might make it very complex to offer appropriate services as patients' needs change, and as local population demographics change, especially in urban areas where there is a high background level of local in and out migration. These local migrations may be more important than international migration, but few studies have investigated the impact of these on mental health.

The limitations to this study are associated with the location as the sample was recruited from the most deprived boroughs in the country and findings might therefore be less applicable to areas with different levels of deprivation. Combining the two sampling techniques aimed to maximise a broad range of individuals with diverse socio-demographic characteristics, but this does preclude looking at the role of each sample source as an influence on the findings. Reassuringly, statistical analyses suggest that the two groups were mostly similar on a number of demographic characteristics (age, gender, occupation, children etc).

The BEMI-I and BEMI-C have produced different type of data on help-seeking and one could interpret this as a weakness in the validity of either method. However, each asked different types of questions, open ended and structured. Although one would expect some overlap in the results, but the findings of each should be seen as complementary tools that can be used to assess the contextual influences in answers given to surveys.

## Conclusion

Our study examined help-seeking for distress in a sample of three ethnic groups. It found that alternative service use was significantly associated with primary care service use similar to seeking help from family. A significant proportion of people did not know how to best resolve their distress, but at the same time most people were active problem solvers and employed a number of strategies to deal with their distress. Our findings support the importance of the community and lay support system, suggesting that services might want to work more closely with traditional healers and families in the community. More mapping of, and engagement with, this sector of health care could improve health promotion and effective treatment.

## Competing interests

The work was supported by a PhD studentship grant from the JRB of Barts and the London, Queen Mary's School of Medicine and Dentistry. KR was recipient of the studentship. She started her appointment at Global Outcomes Research (IPC160), Pfizer Ltd, Sandwich, Kent following her PhD and received no funds to support this work from Pfizer. The author(s) declare they have no further competing interests.

## Authors' contributions

KR coordinated and participated in the data collection, produced the data analyses, drafted the manuscript. KB was grant holder for the study, participated in its design and helped to draft the manuscript. SP participated in the study's design and helped to draft the manuscript. All authors read and approved the final manuscript.

## Pre-publication history

The pre-publication history for this paper can be accessed here:


